# Structural damage identification using single-point vibration data processing

**DOI:** 10.1371/journal.pone.0330909

**Published:** 2025-09-11

**Authors:** Huan-Yi Chu, Meng-Hsuan Tien

**Affiliations:** Department of Power Mechanical Engineering, National Tsing Hua University, Hsinchu, Taiwan; National University of Sciences and Technology, PAKISTAN

## Abstract

Structural health monitoring and damage identification are essential for ensuring the safety and performance of engineering systems. Cracks introduce nonlinear dynamic behavior due to intermittent contact from the opening and closing of crack surfaces, which limits the effectiveness of conventional linear identification methods. Moreover, many existing approaches rely on multiple distributed sensors, which may be impractical in real-world applications. To address these limitations, this study investigates the feasibility of identifying both crack depth and location using single-point vibration measurements. A recently developed nonlinear analysis framework is employed to simulate the dynamic response of a cracked beam, and spectrograms of the tip response under various crack conditions are generated using the short-time Fourier transform. These spectrograms are then used to train a convolutional neural network for damage identification. Numerical results demonstrate that the proposed method achieves high coefficients of determination (R²=0.82–0.97) between the true and identified values for both crack depth and location, provided the training data sufficiently cover damage conditions within the defined parameter ranges. Furthermore, data augmentation is shown to enhance identification accuracy, underscoring the method’s potential for implementation with limited vibration measurements.

## Introduction

The identification of structural damage is critical to avoid catastrophic failure in various mechanical and aerospace systems. To investigate the effects of cracks on structural dynamics, earlier research utilized linear models to analyze how the severity of cracks affects the natural frequencies and mode shapes of a structure [1,2]. However, some research has noted that linear vibration characteristics cannot accurately reflect damage conditions, as cracks do not always remain fully open during vibrations [3,4]. To improve modeling accuracy, breathing-crack models, which account for the opening and closing of crack surfaces, have been developed in many studies to analyze the nonlinear dynamics of cracked structures [5–11]). These studies indicate that nonlinear vibration characteristics can effectively reflect the presence of cracks.

Analytics-based approaches have been widely used to identify the nonlinear dynamics of damaged structures. For example, Chatterjee [12] demonstrated that the ratio of second-order to first-order frequency responses in nonlinear vibrations can be used to estimate structural damage severity. Peng et al. [13] introduced the concept of nonlinear output frequency response functions and applied it to crack detection. Yan et al. [14] used power spectral density analysis to extract the natural frequencies from local free-vibration responses of a cracked beam, thereby quantifying damage severity. Cao et al. [15] proposed a bilinear breathing crack damage identification method to estimate local damage severity in truss structures. Although these methods effectively detect the presence and severity of cracks, they often cannot identify all damage parameters—particularly the crack locations. To address this, several modal-parameter-based methods [16–19] have been developed to estimate crack locations. However, these techniques typically require distributed sensors to measure vibration mode shapes, which may be infeasible in many operational environments.

With the rapid advancement of artificial intelligence, many recent studies have incorporated machine learning techniques into structural damage identification research. For instance, Rosales et al. [20] utilized artificial neural networks to identify damage parameters by analyzing the natural frequencies of beam structures. Nguyen et al. [21] performed a wavelet analysis on response data from multiple measured points and employed deep learning techniques to assess crack conditions. Shirazi et al. [22] used a convolutional neural network (CNN) to extract features from raw vibration response data, enabling the identification of damage parameters for multiple cracks in a composite beam structure. Although these studies have demonstrated the effectiveness of machine learning methods for identifying complete damage parameters, they still rely on modal frequencies and mode shapes as features for classifying crack conditions; hence, multiple sensors are still required.

To eliminate the need for multiple sensors, this paper investigates the feasibility of identifying structural damage parameters by analyzing the single-point vibration response of a cracked cantilever beam. First, the nonlinear dynamics of the cracked beam is numerically modeled using a nonlinear analysis framework proposed by Tien and D’Souza [23]. This framework uses a hybrid symbolic-numerical computational (HSNC) scheme to enable an efficient analysis of the beam’s nonlinear dynamics for a wide range of damage parameters. Subsequently, the short-time Fourier transform (STFT) [24] is employed in this work to extract the frequency content of the computed nonlinear responses at the tip of the beam. The spectrograms corresponding to various combinations of crack depth and location are then fed into a CNN algorithm to construct an identification model for the reverse identification of the damage parameters. Finally, the identification model’s capability to identify both the depth and location of the crack is evaluated and discussed. This study integrates analytical modeling and machine learning to demonstrate the potential of identifying complete damage parameters using vibration response data measured at a single point.

The remainder of the paper is organized as follows. First, the computational framework for computing the nonlinear response of the cracked beam is introduced. The CNN algorithm for constructing the identification model is then presented. Subsequently, the results of identifying damage parameters through single-point vibration response analysis are discussed. Finally, the conclusion and discussion are presented, and future research directions are outlined.

## Nonlinear analysis framework

**Fig 1:**
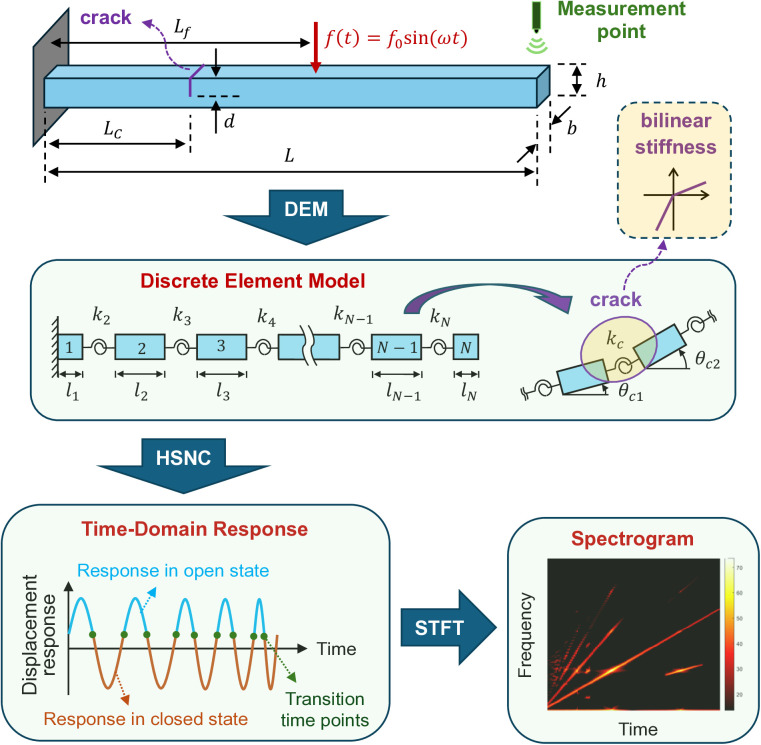
Nonlinear analysis framework.


This section introduces the nonlinear analysis framework [23], as illustrated in Fig 1, for analyzing the dynamics of a cracked cantilever beam. The beam structure is first modeled using the discrete element method (DEM) [25], which discretizes the beam into *N* blocks. For each block i, the moment of inertia is defined as $J_{i}=\rho bhl_{i}\left(h^{2}+{l_{i}}^{2}\right)/12$, where ρ, b, and h represent the density, width, and height of the beam, and $l_{i}$ represents the length of the i−th block. Adjacent blocks are connected by rotational springs with stiffness $k_{i}=EI/l_{i}$, where E and Irepresent the Young’s modulus and the second moment of area, respectively. The equation of motion of the beam can be expressed as follows:

$$\begin{array}{c} {𝐉}^{\prime }\ddot{\Theta }+{𝐂}^{\prime }(\Theta )\dot{\Theta }+{𝐊}^{\prime }(\Theta )\Theta =𝐟\prime (t), \end{array}$$ (1)

where ${𝐉}^{\prime }$, ${𝐂}^{\prime }$, and ${𝐊}^{\prime }$ are the moment of inertia, damping, and stiffness matrices, respectively; Θ represents the coordinate vector; and 𝐟′(t) represents the forcing vector. Detailed derivations of these matrices and vectors are provided in [25] and detailed in Appendix. To model the breathing phenomenon at the crack, a bilinear stiffness model is applied at the cracked section:


$$\begin{array}{c} k_{c}=\left\{ \begin{array}{c} \frac{EI}{l_{c}}\text{when crack is closed}\left(\theta_{c1}-\theta_{c2}\leq 0\right)\\ \frac{1}{\frac{l_{c}}{EI}+\frac{72}{Ebh^{2}}H(r)}\text{when crack is open}(\theta_{c1}-\theta_{c2}>0) \end{array}\right. \end{array}$$ (2)

where $l_{c}$ is the length of the block at the cracked section, and H(r) is an empirical function describing stiffness reduction when the crack opens, as indicated by the following [26]:


$$H(r)=1.98r^{2}-3.277r^{3}+14.43r^{4}-31.26r^{5}+63.56r^{6}$$

$$\begin{array}{c} -103.36r^{7}+147.52r^{8}-127.69r^{9}+61.50r^{10} \end{array}$$ (3)

where r=d/h in Eq. (3) represents the crack depth ratio. By integrating Eq. (2) into Eq. (1), the motion equation for the cracked beam is established as a bilinear system:


$${𝐉}^{\prime }{\ddot{\Theta }}_{c}(t)+{{𝐂}_{c}}^{\prime }{\dot{\Theta }}_{c}(t)+{{𝐊}_{c}}^{\prime }\Theta_{c}(t)=𝐟(t)$$

$$\begin{array}{c} {𝐉}^{\prime }{\ddot{\Theta }}_{o}(t)+{{𝐂}_{o}}^{\prime }{\dot{\Theta }}_{o}(t)+{{𝐊}_{o}}^{\prime }\Theta_{o}(t)=𝐟(t) \end{array}$$ (4)

where the subscript c and o represent the closed and the open states of the cracked beam, respectively. Note that the dynamic characteristics of Eq. (4) have been experimentally validated in [25].

The HSNC method proposed in [23] is then used to compute the response of Eq. (4). HSNC expresses the response of the cracked beam in each state using analytical modal solutions. For underdamped modes, these solutions can be expressed as follows:


$$\begin{array}{c} q_{c,j}(t)=A_{c,j}e^{{-\zeta }_{c,j}\omega_{c,j}t}\text{sin}\left(\omega_{cd,j}t+\phi_{c,j}\right)+X_{c,j}\text{sin}\left(\omega t+\phi^{*}-\Phi_{c,j}\right) \end{array}$$

$$\begin{array}{c} q_{o,j}(t)=A_{o,j}e^{{-\zeta }_{o,j}\omega_{o,j}t}\text{sin}\left(\omega_{od,j}t+\phi_{o,j}\right)+X_{o,j}\text{sin}\left(\omega t+\phi^{*}-\Phi_{c,o}\right) \end{array}$$ (5)

where $q_{c,j}$ and $q_{o,j}$ represent the j−th modal displacement in the closed state and open state, respectively; $A_{c,j}$ and $A_{o,j}$ are transient response amplitudes; $\zeta_{c,j}$ and $\zeta_{o,j}$ are damping ratios; $\omega_{c,j}$ and $\omega_{o,j}$ are modal frequencies; $\omega_{cd,j}$ and $\omega_{od,j}$ are damped frequencies; $\phi_{c,j}$ and $\phi_{o,j}$ are phase angles of the transient responses; $X_{c,j}$ and $X_{o,j}$ are steady-state response amplitudes; ω is the excitation frequency; $\phi^{*}$ is the phase angle of the harmonic excitation; and $\Phi_{c,j}$ and $\Phi_{o,j}$ are phase angles of the steady-state responses. Note that the parameters $A_{c,j}$, $A_{o,j}$, $\phi_{c,j}$, and $\phi_{o,j}$ in Eq. (5) depend on the initial conditions at the transition time points where the system switches between the states. To obtain these parameters, the transition points and their associated displacements and velocities are computed using a numerical incremental search process [23]. The temporal history of the entire nonlinear response is then constructed by combining the analytical solutions of each linear state. The HSNC method enables efficient dynamic simulation for cracked beams and can be used to study the effects of damage parameters on the nonlinear dynamic response [27,28]. Note that the HSNC method has been numerically validated in [23]. Finally, STFT analysis [24] is applied to the beam’s tip response to extract frequency contents under various crack conditions. The STFT spectrogram samples are then used to train a damage identification model using a machine learning algorithm.

### The construction of identification models

**Fig 2:**
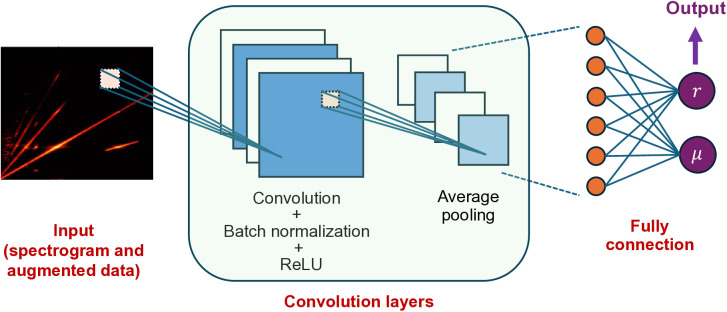
CNN structure.

To generate training data, the STFT spectrograms corresponding to various crack conditions are first computed using the nonlinear analysis framework. The crack condition is defined using the crack depth ratio r=d/h and the crack location ratio $\mu =L_{c}/L$. Each spectrogram sample is stored in a matrix format and is labeled by the corresponding damage parameters (r,μ). As shown in Fig 2, after computing the STFT spectrograms for all considered damage parameter values, the resulting spectrogram samples are fed into a CNN framework [29] to train a damage identification model. Note that data augmentation can be performed on spectrogram data if the training set has insufficient samples. In the training process, multiple convolutional layers are used to extract features from the input data. In each of the convolution layers, batch normalization is performed to avoid network overfitting and to accelerate the training process, and the rectified linear unit (ReLU) is used to introduce nonlinearity into the network. Following each convolution layer, an average pooling layer is employed to reduce the dimensionality of the feature maps, which helps prevent overfitting and reduces the computational cost. Finally, a fully connected layer is used to provide the final identification results. In this study, the output layer has two labels, namely r and μ. The machine learning is conducted using the MATLAB Deep Learning Toolbox.

### Identification results

In this study, a cantilever beam with length L=0.55m, width b=0.026m, and height h=0.026mis discretized into 100 blocks (N=100) using the DEM. The Young’s modulus and density are set to E=60.62GPa and ρ=2785.18 kg/$m^{3}$, respectively. The Rayleigh damping ${𝐂}^{\prime }=\gamma {𝐊}^{\prime }$, with $\gamma =7.45{\times 10}^{-5}$, is used to model the damping property of the structure. A harmonic force f(t)=15sin(αttextrmNis applied at a distance of $L_{f}=0.027$m from the fixed end, where α represents the excitation frequency that can vary in the numerical analysis.

**Fig 3:**
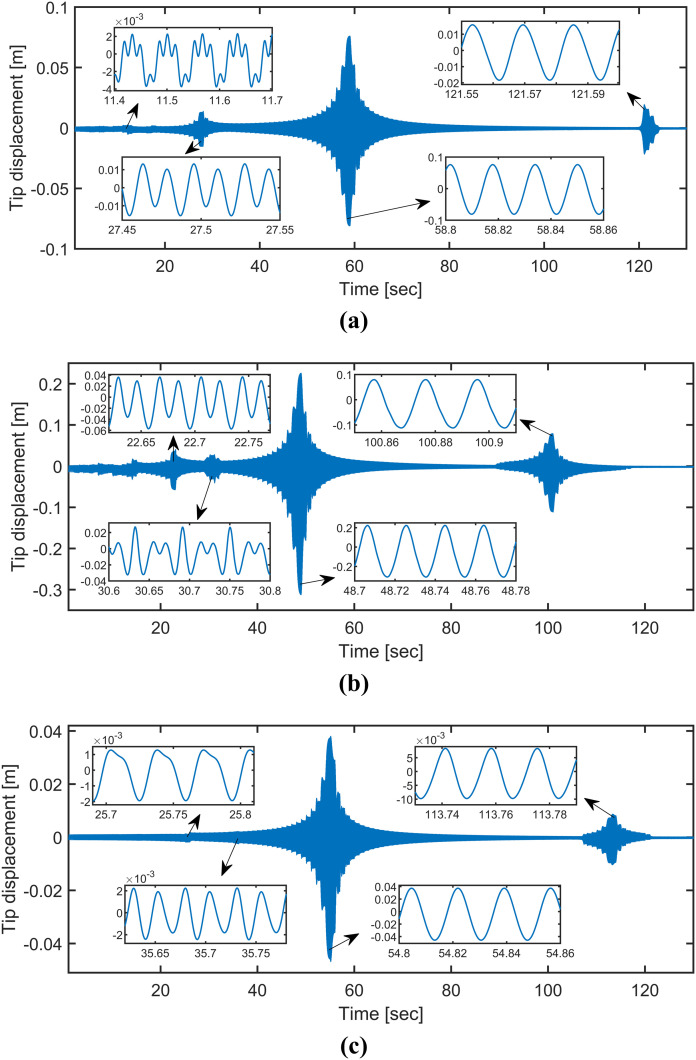
Tip responses for (a) (𝐫,μ)=(0.3,0.0055), (b) (𝐫,μ)=(0.6,0.0055), and (c) (𝐫,μ)=(0.6,0.2727).

To investigate the nonlinear dynamic features of the cracked beam, an upward stepped frequency sweep excitation with a rate of $1\frac{\text{Hz}}{\text{Sec}}$ is applied on the structure for the following three damage parameter sets: (1) (r,μ)=(0.3,0.0055), (2) (r,μ)=(0.6,0.0055), and (3) (r,μ)=(0.6,0.2727). The frequency sweep spans the range of 5Hz to135Hz, covering up to twice the system’s first natural frequency of 64.75Hz. The displacement responses at the beam’s tip corresponding to these damage parameter sets are computed using HSNC and are presented in Fig 3. The results show that the responses exhibit distinct harmonic patterns depending on the damage parameters, as highlighted in the insets.

**Fig 4:**
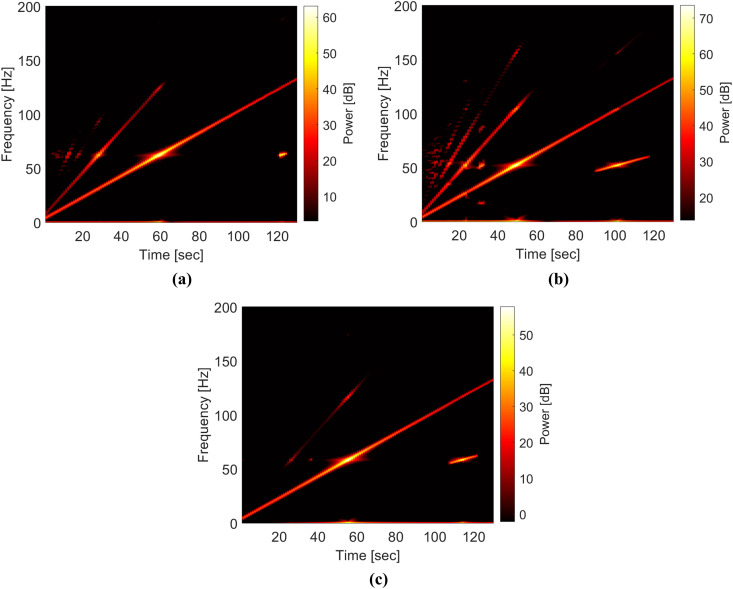
STFT spectrograms of tip response for (a) (𝐫,μ)=(0.3,0.0055), (b) (𝐫,μ)=(0.6,0.0055), and (c) (𝐫,μ)=(0.6,0.2727). The longest strips in these spectrograms reflect the excitation frequency.

Next, as shown in Fig 4, the STFT spectrograms of the tip responses corresponding to the three damage parameter sets are computed to analyze their frequency components. In each spectrogram, the longest stripe reflects the frequency component matching the excitation frequency. Among the three cases, the response for (r,μ)=(0.6,0.0055), shown in Fig 4(b), exhibits the strongest nonlinearity, as evidenced by the presence of multiple super-harmonic components early in the sweep and a distinct subharmonic response near the end of the frequency range. When the crack depth ratio is reduced to r=0.3 (i.e., case (r,μ)=(0.3,0.0055)), the subharmonic response nearly disappears, although several super-harmonic components remain visible, as presented in Fig 4(a). In contrast, increasing the crack location ratio to μ=0.2727 (i.e., case (r,μ)=(0.6,0.2727)) leads to the disappearance of most super-harmonic components, while the subharmonic response persists, as shown in Fig 4(c). These trends in super-harmonic and subharmonic behavior are consistent with observations reported in previous studies [27,30]. Overall, the variations in frequency content, as illustrated by the spectrogram features, are closely linked to the damage parameters and serve as key indicators for identifying damage characteristics in this study.

Subsequently, to assess the feasibility of identifying damage parameters using the spectrogram of tip response, the response of the beam is computed for various combinations of crack depth r and crack location μ using the nonlinear analysis framework. The considered ranges for crack depth and crack location are 0≤r≤0.5 and 0≤μ≤0.91, respectively. Within these intervals, uniformly distributed values of r and μ are selected to generate the spectrogram samples. As shown in Table 1, the crack parameters are discretized into three groups of distributions. In group (1), 11 uniformly distributed values of r and 11 uniformly distributed values of μ were selected to generate spectrogram data, yielding 121 spectrogram samples, each corresponding to a unique combination of (r,μ). In group (2), 26 uniformly distributed values of r and 26 uniformly distributed values of μ were selected, resulting in 676 spectrogram samples. In group (3), 51 uniformly distributed values of r and 51 uniformly distributed values of μ were selected, producing a total of 2,601 spectrogram samples. The proposed CNN algorithm was then used to build identification models using the spectrogram samples in groups (1), (2), and (3). Note that this yields three distinct damage identification models. In the CNN training process, 85% of the spectrogram samples from each group were randomly selected for training, and the remaining 15% samples were used for validation. The proposed CNN architecture used 7 convolution layers, and the average pooling step in each layer used a pooling area of 2×2 and a stride of 2. The training times for the models based on groups (1), (2), and (3) are 7.1 seconds, 28.6 seconds, and 122.8 seconds, respectively. All training was conducted on a workstation equipped with an Intel i7 processor (2.9 GHz), an NVIDIA GeForce GTX 1650 GPU, and 64 GB of memory.

**Table 1:** Selection of r and μ for spectrogram data generation.

Group	Number of r	Number of μ	Total parameter combinations	Number of parameter combinations for training (85%)	Number of parameter combinations for validation (15%)
(1)	11	11	11 × 11=121	103	18
(2)	26	26	26 × 26=676	575	101
(3)	51	51	51 × 51=2,601	2,211	390

**Fig 5:**
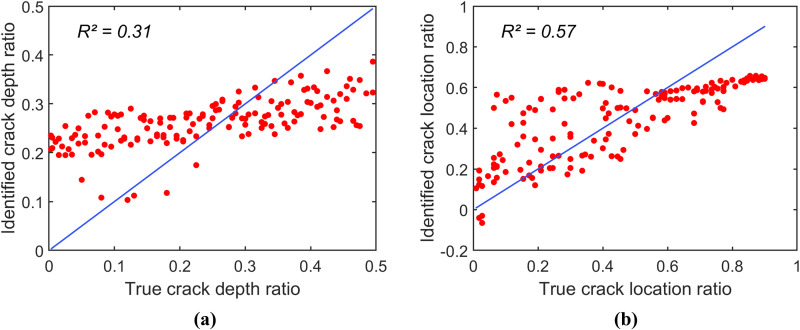
Scatter plots of prediction for the identification model constructed using spectrogram samples in group (1). (a) Scatter plot for r. (b) Scatter plot for μ.

**Fig 6:**
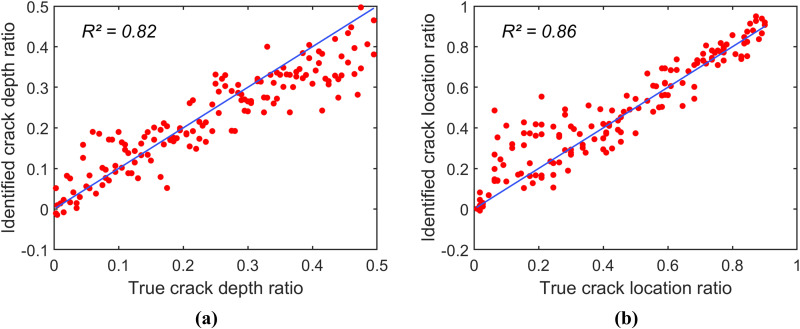
Scatter plots of prediction for the identification model constructed using spectrogram samples in group (2). (a) Scatter plot for r. (b) Scatter plot for μ.

**Fig 7:**
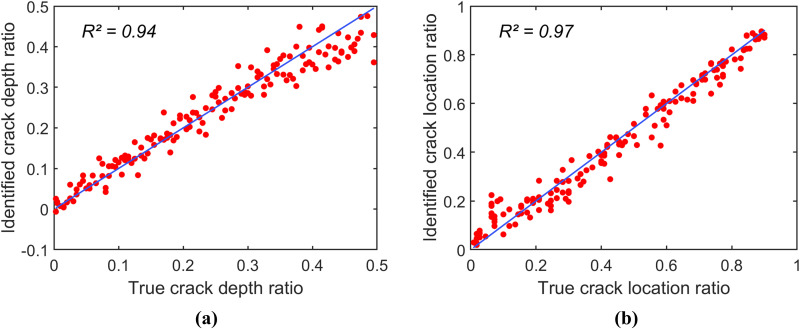
Scatter plots of prediction for the identification model constructed using spectrogram samples in group (3). (a) Scatter plot for r. (b) Scatter plot for μ.

To investigate the identification accuracy of each CNN-identification model, these models are applied to reversely identify the crack depth ratio r and the crack location ratio μ of spectrogram samples in a testing data set. Notably, this testing data set includes spectrogram samples corresponding to 150 combinations of (r,μ) that are not included in the training and validation data sets. Figs 5–6, and 7 present the scatter plots of the identification results of the models constructed using the spectrogram samples in the groups (1), (2), and (3), respectively. Note that all data points in a scatter plot represent the relationship between the true values and the identified values of the parameter; thus, the identification accuracy of the identification models can be determined by how closely the scatter plot follows the line of equality (i.e., the blue line in the scatter plots).

Fig 5 shows that the identification model constructed using the data in group (1) exhibits poor accuracy. The coefficients of determination ($R^{2}$) for the crack depth ratio and crack location ratio are 0.31 and 0.57, respectively. This identification model, based on only 121 spectrogram samples, is insufficient for accurately identifying the damage parameters. In contrast, Figs 6 and 7 illustrate that the identification accuracy improves significantly as the spectrogram samples are expanded to include finer grids of (r,μ). The model constructed using data in group (2) has the coefficients of determination ($R^{2}$) values of 0.82 and 0.86 for the crack depth ratio and the crack location ratio, respectively. The model constructed using data in group (3) achieved even higher $R^{2}$ values of 0.94 and 0.97 for the damage parameters. Overall, Figs 4–6 demonstrate that the spectrogram response provides useful features for identifying damage parameters, provided the training and validation data include a sufficiently dense grid for (r,μ).

**Fig 8:**
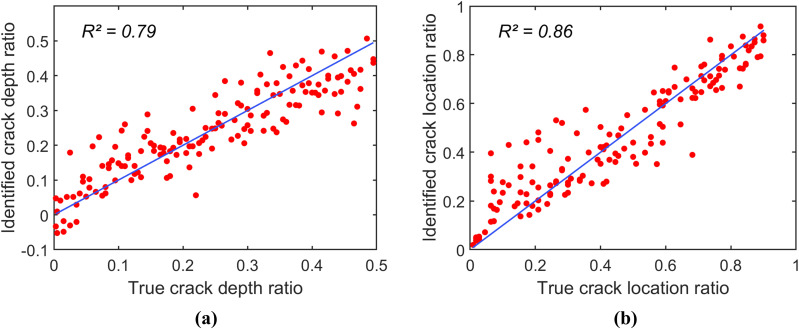
Scatter plots of prediction for the identification model constructed using augmented spectrogram samples in group (1). (a) Scatter plot for r. (b) Scatter plot for μ.

Finally, data augmentation is applied to the spectrogram samples in group (1) to evaluate whether it can improve the CNN model’s identification accuracy when measured spectrogram samples are limited. In this study, rotation, scaling, and translation techniques [31] are applied to the training set of group (1), increasing the number of samples from 103 to 2,575. Fig 8 presents the scatter plots of the identification model constructed using the augmented spectrogram samples. The results demonstrate that data augmentation significantly enhances identification accuracy, with R^2^ values for the crack depth ratio and crack location ratio rising to 0.79 and 0.86, respectively. The total CPU time required for data augmentation and model training is 268.4 sec.

## Conclusion and discussion

In this study, an efficient nonlinear analysis framework and a CNN machine learning model are integrated to demonstrate the feasibility of identifying structural damage parameters through single-point vibration analysis. First, the nonlinear analysis framework is employed to compute the nonlinear dynamic responses of a cantilever beam with various crack conditions. Subsequently, STFT is applied to the computed responses to obtain spectrograms for various crack parameters, and these spectrogram samples are divided into three sets—a training set, a validation set, and a testing set. The spectrogram samples in the training and validation sets are then fed into the proposed CNN model to construct damage identification models. Finally, the spectrogram samples in the testing set are used to test the prediction accuracy of the identification models.

Numerical results demonstrate that the proposed system identification approach can successfully identify both crack depth and crack location using vibration responses measured at the beam’s tip. The coefficients of determination between the actual and predicted damage parameters range from 0.82 to 0.97 when the identification model is trained with 575 and 2,211 spectrogram datasets. These datasets are generated using 26 and 52 uniformly distributed values of the crack depth ratio *r* and the crack location ratio *µ*, respectively. This result highlights the effectiveness of the proposed method when the training data encompass sufficiently diverse damage parameter values within the defined ranges. Furthermore, data augmentation is shown to significantly improve identification accuracy. The coefficients of determination for (*r*, *µ*) increase from (0.31, 0.57) to (0.79, 0.86) after applying rotation, scaling, and translation techniques to the original training set of 103 spectrogram datasets.

This study outlines a potential future research direction in structural damage identification using a single-point vibration measurement approach. Unlike conventional methods, the proposed method does not require mode shape information, thereby eliminating the need for multiple sensors. The identification strategy is particularly well-suited for engineering systems subjected to frequency sweep excitation—such as rotating machinery—as it leverages vibration response data across a frequency range for both training and prediction. This approach could be implemented during a rotor’s power-up or power-down phases, during which broadband vibration data are naturally generated. Nevertheless, further investigation is needed to adapt and validate the method for specific system applications.

Below are key challenges for future work:

(1) In this study, the influence of vibration measurement noise on the accuracy of damage identification was not considered. However, in practical applications, sensor noise and environmental disturbances are inevitable and may affect identification performance. Future work should incorporate simulated or experimentally measured noise into the training and validation processes to enhance model robustness.
(2) The identification models in this study are constructed using spectrogram samples generated from a wide range of crack depth and location combinations (r,μ). To reduce the amount of required data and measurements, future research should investigate strategies for selecting key damage parameter values—such as sensitivity analysis or optimization-based sampling methods—to efficiently train accurate identification models.
(3) The effectiveness of alternative machine learning techniques remains unexplored. Future studies should evaluate how other approaches affect identification accuracy.
(4) The nonlinear modeling approach employed in this study is limited to characterizing two crack parameters: depth and location. Other parameters, such as crack angle, orientation, width, and material property variations, may also influence system dynamics and identification accuracy. Future research should explore integrating the proposed machine learning framework with more comprehensive nonlinear models capable of capturing these additional parameters, thereby enabling multi-parameter identification from single-point measurements.

## Appendix

The moment of inertia matrix ${𝐉}^{\prime }$, the stiffness matrix ${𝐊}^{\prime }$, and the forcing vector ${𝐟}^{\prime }$ in Eq. (1) can be expressed as


$${𝐉}^{\prime }=𝐉+\frac{\text{1}}{\text{4}}𝐋𝐁{𝐀}^{-\text{1}}𝐌{\left({𝐀}^{\text{T}}\right)}^{-\text{1}}{𝐁}^{\text{T}}𝐋$$ (6)

$${𝐊}^{\prime }=𝐀𝐊{𝐀}^{\text{T}}$$ (7)

$${𝐟}^{\prime }={f(t)𝐥}^{\prime }$$ (8)

where $𝐉=\text{diag}(J_{2},J_{3},\cdots ,J_{N})$, $𝐌=\text{diag}(m_{2},m_{3},\cdots ,m_{N})$, and $𝐊=\text{diag}(k_{2},k_{3},\cdots ,k_{N})$ are diagonal matrices. f(t) represents the harmonic force. 𝐋 and ${𝐥}^{\prime }$ are the block length matrix and length vector that can be expressed as $𝐋=\text{diag}(l_{2},l_{3},\cdots ,l_{N})$ and ${𝐥}^{\prime }={\left[l_{2},l_{3},\cdots ,l_{k},0,\cdots ,0\right]}^{\text{T}}$, where the subscript k denotes the index of the block to which the force is applied. 𝐀 and 𝐁 can be expressed as


$$𝐀=[\begin{array}{ccccccc} -1 & 1 & 0 & \cdots & 0 & 0 & 0\\ 0 & -1 & 1 & \cdots & 0 & 0 & 0\\ \vdots & \vdots & \vdots & \ddots & \vdots & \vdots & \vdots \\ 0 & 0 & 0 & \cdots & 0 & -1 & 1\\ 0 & 0 & 0 & \cdots & 0 & 0 & -1 \end{array}]\left(N-1)\times (N-1\right)$$ (9)

$$𝐁=[\begin{array}{ccccccc} 1 & 1 & 0 & \cdots & 0 & 0 & 0\\ 0 & 1 & 1 & \cdots & 0 & 0 & 0\\ \vdots & \vdots & \vdots & \ddots & \vdots & \vdots & \vdots \\ 0 & 0 & 0 & \cdots & 0 & 1 & 1\\ 0 & 0 & 0 & \cdots & 0 & 0 & 1 \end{array}]\left(N-1)\times (N-1\right)$$ (10)
